# The prognostic value of biomarkers in stroke

**DOI:** 10.1186/s12979-016-0074-z

**Published:** 2016-05-31

**Authors:** Francesco Iemolo, Enzo Sanzaro, Giovanni Duro, Antonello Giordano, Maurizio Paciaroni

**Affiliations:** Institute of Biomedicine and Molecular Immunology (IBIM), National Research Council, Palermo, Italy; Department of Neurology, “R. Guzzardi” Hospital-ASP Ragusa, Via Papa Giovanni XXIII, Vittoria (Ragusa), Italy; Department of Neuroscience, University of Catania, Catania, Italy; Stroke Unit and Division of Cardiovascular Medicine, University of Perugia, Perugia, Italy

## Abstract

**Background:**

Ischemic injury triggers inflammatory cascades and changes in the protein synthesis, neurotransmitters and neuro-hormones in the brain parenchyma that may further amplify the tissue damage. The “Triage® Stroke Panel”, a biochemical multimarker assay, detects Brain Natriuretic Peptide (BNP), D-Dimers (DD), Matrix-Metalloproteinase-9 (MMP-9), and S100β protein generating a Multimarker index of these values (MMX). The aims of this prospective study in consecutive patients with ischemic or hemorrhagic stroke were to assess: 1) the rate of an increase of biomarkers (BNP, D-dimer, MMP-9 and S-100β) tested with the Triage Stroke Panel; 2) the correlation between the increase of these biomarkers and functional outcome at 4 months; 3) the risk factors for the increase of biomarkers.

**Methods:**

The outcome of the study was 120-day mortality and it was compared in patients with Stroke Panel >4 and ≤4. Multiple logistic regression analyses were performed to identify independent predictors for death and for the increase of biomarkers.

**Results:**

244 consecutive patients (mean age 73.02 years; 53.7 % males) were included in the study; 210 ischemic strokes and 34 hemorrhagic strokes. 161/244 (66.0 %) had an increase of biomarkers. At 120 days, 85 patients had died (34.8 %). Death was seen in 68/161 patients with an increase of biomarkers (42.2 %) compared with 17/83 patients without (20.5 %). Regression logistic analysis found that a Stroke Panel >4 (OR 3.1; 95 % CI 1.5–6.2, *p* = 0.002) was associated with mortality. The increase of biomarkers was independently predicted by an increase of PCR on admission (OR 2.9, 95 CI 1.4–6.0, *p* = 0.003).

**Conclusions:**

An increase of biochemical markers such as BNP, D-Dimers, MMP-9, and S100β tested with a Triage Stroke Panel (>4) was correlated with mortality at 120 days from stroke onset.

## Background

Ischemic injury triggers inflammatory cascades and changes in the protein synthesis, neurotransmitters and neuro-hormones in the brain parenchyma that may further amplify tissue damage. Inflammation is a key event in this ischemic cascade after cerebral ischemia and some inflammatory-related molecules are produced and secreted in the neurovascular unit, which could activate immune system in response to cell damage [1]. Inflammatory markers to predict outcome would be useful in the management of ischemic stroke patients as potential surrogate measures. This is supported by studies showing elevated inflammatory markers and is associated with final infarct volume, including TNF-α, IL-6, ICAM-1, MMP-2 and MMP-9 [2]. Matrix metalloproteinase-9 (MMP-9) belongs to a family of zinc-binding proteolytic enzymes that are capable of degrading all components of the extracellular matrix including collagen IV, laminin and fibronectin. Cerebral MMP-9 expression has been found to increase in the early hours after stroke onset and MMP-9 is thought to be a mediator of increased blood–brain barrier permeability and hemorrhagic transformation following ischemic stroke [3].

Other biomarker elevations have been associated with stroke such as Brain Natriuretic Peptide (BNP), D-dimer and S-100β protein. BNP is a neuro-hormone that has potent natriuretic, diuretic and vasodilatatory effects. Elevations of the circulating BNP concentration have been observed in patients who experienced delayed ischemic neurological deficits due to cerebral vasospasm following subarachnoid hemorrhage [4]. D-dimer is a cross-linked fibrin degradation product that is released into the circulation during fibrinolysis. D-dimer elevations have been observed following ischemic stroke and may be indicative of stroke progression [5]. S-100β is a calcium-binding protein that is found in high concentration within glial cells. Elevations of the circulating S-100β concentration have been observed following ischemic stroke and intracranial hemorrhage, and are associated with infarct size [6, 7].

The Triage Stroke Panel is a rapid, point-of-care fluorescence immunoassay to be used with the Triage MeterPlus for the rapid, quantitative measurement of BNP, fibrin degradation products containing D-dimer, MMP-9 and S-100β in EDTA-anticoagulated whole blood or plasma specimens. The test utilizes a proprietary algorithm for the automatic calculation of a single Multimarker Index (MMX) result from the individual biomarker values. The MMX result is used as an aid in the assessment and diagnosis of stroke [8, 9].

The aims of this prospective study in consecutive patients were to assess: 1) the rate of an increase of biomarkers (BNP, D-dimer, MMP-9 and S-100β) tested with Triage Stroke Panel in patients admitted for ischemic or hemorrhagic stroke; 2) the correlation between the increase of these biomarkers and functional outcome at 120 days; 3) the risk factors for the increase of biomarkers. This work came from daily hospital activity, in which patients underwent various medical tests. For this reason, we did not require permission from our institution’s Ethics Committee. All patients provided informed consent before participation in the study.

## Methods

Consecutive patients admitted to the Department of Neurology of Vittoria Hospital (Italy) with objectively diagnosed stroke between January 1st, 2006 and April 15th, 2009 were included in this prospective cohort study. Patients with transient ischemic attack, cerebral venous thrombosis and subarachnoid hemorrhage on admission were excluded. All patients were assessed by a neurologist to determine the diagnosis of stroke (neurological deficit lasted >24 h) and its pathological and etiological subtypes [10]. On admission, stroke severity was assessed using the NIH (National Institute of Health) Stroke Scale. The NIHSS is a clinical assessment tool that is widely used in clinical trials and practice to evaluate stroke-related neurological deficits. It assesses speech, language cognition, inattention, visual field abnormalities, motor and sensory impairments, and ataxia. The maximum possible score, in case of severe stroke, is 42.

The study centre provided stroke unit standard care for each patient. All patients were monitored for blood pressure, temperature, glucose level, heart rate and blood gases in the first days after stroke.

In all patients cerebral Computed Tomography (CT) examination without contrast was performed on admission and after 48–72 h from stroke onset to define both topography and extension of the ischemic or hemorrhagic lesion. Patients were admitted to the hospital within 12 h from the stroke onset.

Based on standard templates the size of the lesion was quantified as: a) lacunar (lesion in the anterior or posterior circulation <1.5 cm), b) non lacunar (lesion in the anterior or posterior circulation >1.5 cm).

### Stroke risk factors

Data was collected on stroke risk factors: age, gender, history of hypertension (blood pressure of >140/90 mmHg at least twice before stroke or already under treatment with antihypertensive drugs), history of diabetes mellitus (glucose level >126 mg/dL preprandial on 2nd examination, glucose level >200 mg/dL postprandial, or HbA_1c_ > 8.5 %, or under hypoglycemic treatment), current cigarette smoking, history of hyperlipidemia (total cholesterol concentration >200 mg/dL and/or triglyceride concentration >140 mg/dL the day after admission or already under lipid lowering therapy), history of symptomatic ischemic heart disease (proven myocardial infarction, history of angina or existence of multiple lesions on thallium heart isotope screen or evidence of coronary disease on coronary angiography), atrial fibrillation, or previous stroke/transient ischemic attacks (TIAs). Other baseline variables that were obtained at admission for each patient included homocysteinemia (normal values ≤15 μmol/l) and PCR (normal values ≤10 mg/dL).

All cases had ultrasonography examination of the carotid and vertebral arteries. The degree of stenosis on ultrasonography was evaluated on a duplex sonography with power Doppler imaging; linear stenosis, area stenosis and peak systolic velocity (PSV) were measured in the most stenotic part of the ICA or vertebral artery [11, 12]. Patients were considered to have carotid atherosclerosis if at least one vessel had stenosis ≥50 % or atherosclerotic occlusion. Occlusion of an artery was defined as an absence of flow and when there was the presence of a visible plaque, it was diagnosed to be caused by atherosclerosis.

### Evaluation of outcome

Patients were followed up prospectively by face-to face or telephone interview. Study outcome was 120-day mortality. The time of occurence and the cause of death were recorded. The causes of death were divided into: neurological (recurrence of stroke, status epilepticus, edema, herniation), cardiovascular (myocardial infarction, heart failure, sudden death, other cardiovascular disease) and other causes (pneumonia, cancer, pulmonary embolus and other causes).

### Measurement of biomarkers with Triage Stroke Panel

The analysis of the biochemical markers (BNP, D-Dimers, MMP-9, S 100 B, MMX) was done by Sandwich-Fluorescence-Immunoassay-Technology of the so-called Triage® Stroke Panel using blood from an EDTA sample taken within 15 min after admission.

#### Principles of the test procedure

The test procedure involves the addition of several drops of an EDTA whole blood or plasma specimen to the sample port on the Test Device. After addition of the sample, the cells are automatically separated from the plasma via a filter contained in the Test Device. The sample reacts with fluorescent antibody conjugates within the reaction chamber and flows down the Test Device detection lane by capillary action. Complexes of each fluorescent antibody conjugate are captured on discrete zones resulting in binding assays that are specific for each biomarker. The Test Device is inserted into the Triage MeterPlus (hereafter referred to as Meter) and results are measured and displayed on the screen. These results are then presented as a single composite MMX result that is calculated automatically. This composite MMX result is the test result that is used as an aid in the diagnosis and assessment of ischemic stroke [9]. All analyte concentrations and the MMX result are stored in the Meter memory and are available on demand. The MMX result reportable range is 0 to 10. The manufacturer’s recommended cutoffs are 1.3 and 5.9. Using these cutoffs the interpretation is as follows: MMX results less than or equal to 1.3 represent a low probability of stroke; MMX results greater than 1.3 are considered abnormal and suggest the need for further evaluation; MMX results greater than 5.9 represent a high probability of stroke. However, it is recommended that each laboratory should determine if these cutoffs are appropriate for the patient population that is to be evaluated. We have used a score of > 4 because our statistical analysis detects that values higher than 4 correspond with a greater degree of disability and increased mortality.

### Statistical analysis

A receiver operating characteristic (ROC) curve was applied to determine a cut-off point of the panel that could best predict the occurrence of death within 120 days. After the determination of cut-off point, outcomes in patients with Stroke Panel >4 and ≤4 were compared by Χ^2^ test. The first step of analysis was aimed at identifying predictors of adverse outcome (death) at 120 days including biomarkers tested with Triage Stroke Panel. Univariate tests were used to compare clinical characteristics on admission, preexisting risk factors for stroke and CT findings in comparison to patients who died. All variables (age, sex, vascular risk factors, stroke severity on admission, PCR and Stroke Panel > 4) were subjected to multiple logistic regression analysis to identify independent predictors for death.

The second step of analysis was aimed at identifying predictors of the increase of biomarkers (Stroke Panel > 4) among baseline findings. Univariate tests were applied to compare clinical characteristics on admission, preexisting risk factors for stroke and CT findings to patients with or without an increase of biomarkers. All variables were subjected to multiple logistic regression analysis to identify independent predictors for the increase of biomarkers.

Data was analyzed with the SPSS/PC Win package 19.0 [13].

## Results

### Incidence of an increase of biomarkers (Stroke panel > 4)

244 consecutive patients (mean age 73.02 years; 53.7 % males) were included in the study; 210 patients had ischemic stroke and 34 patients hemorrhagic stroke. Among 244 patients included in the analysis, 161 (66.0 %) had an increase of biomarkers.

### Influence of an increase of biomarkers on adverse outcome

At 120 days, 85 patients had died (34.8 %). The causes of death were neurological in 21 patients, cardiovascular in 9 patients, other causes in 4 and unknown in 51 patients. In these last cases, by interviewing family members and examining clinical documents, it has not been possible to obtain adequate information about the causes of death. In Table 1, the characteristics of the patients alive and deceased are summarized.

**Table 1:** Characteristics of the deceased or alive patients at 120 days

Risk factors	Total (*n* = 244)	Alive patients (*n* = 159)	Deceased patients (*n* = 85)	*P*
Age (mean)	73.02 ± 13.39	72.66 ± 14.63	73.69 ± 10.73	0.5
Sex male	131 (53.7 %)	87 (54.7 %)	44 (51.8 %)	0.6
Hypertension	155 (63.5 %)	98 (61.6 %)	57 (67.1 %)	0.4
Diabetes mellitus	71 (29.1 %)	48 (30.2 %)	23 (27.1 %)	0.6
Hypercholesterolemia	37 (15.2 %)	26 (16.3 %)	11 (12.9 %)	0.5
Hypertrygliceridemia	16 (6.5 %)	11 (6.9 %)	5 (5.9 %)	1.0
Current smoking	20 (8.2 %)	12 (7.5 %)	8 (9.4 %)	0.6
TIA history	8 (3.3 %)	5 (3.1 %)	3 (3.5 %)	1.0
Stroke recurrence	44 (18.0 %)	31 (19.5 %)	13 (15.3 %)	0.4
Ischemic heart disease	69 (28.3 %)	40 (25.1 %)	29 (34.1 %)	0.08
Atrial fibrillation	10 (4.1 %)	6 (3.8 %)	4 (4.7 %)	0.7
Carotid atherosclerosis	27 (11.1 %)	19 (12.0 %)	8 (9.4 %)	0.6
Ischemic stroke (qualifying event)	210 (86.1 %)	140 (88.1 %)	70 (82.3 %)	0.2
Non-lacunar stroke	120 (49.2 %)	73 (45.9 %)	47 (52.3 %)	0.04
NIHSS on admission (mean)	8.78 ± 6.22	7.79 ± 5.92	10.14 ± 6.37	0.001
Hyperhomocysteimenia	106 (43.4 %)	65 (40.9 %)	41 (48.2 %)	0.2
PCR increase	98 (40.2 %)	51 (30.1 %)	47 (55.3 %)	0.001
Stroke Panel >4	161 (66.0 %)	93 (58.5 %)	68 (80.0 %)	0.001

**Fig. 1:**
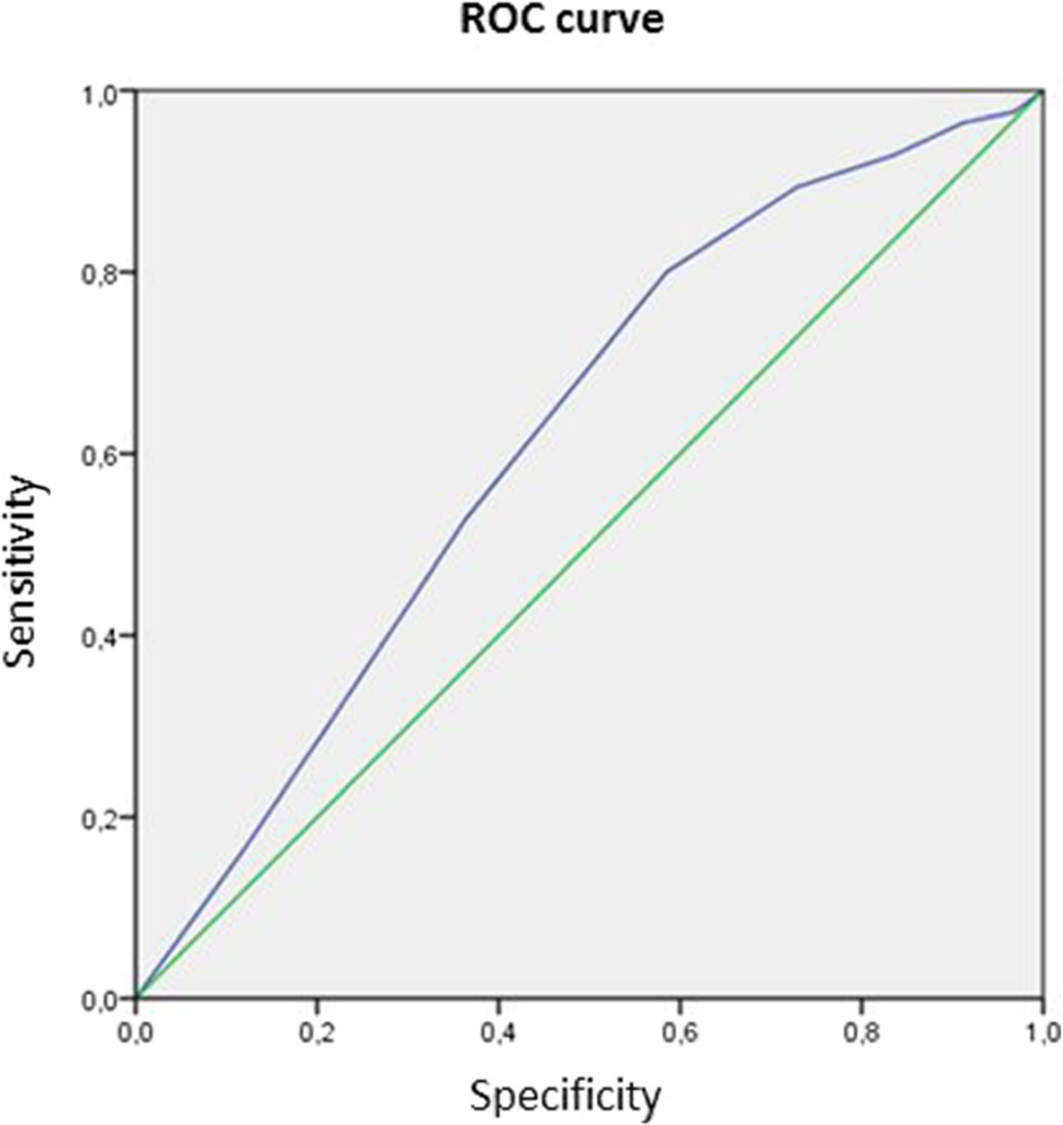
a Stroke Panel >4 predicted the occurrence of dead at 120 days; area under the curve 0.62 (95 % CI 0.55–0.69), *p* = 0.002

The ROC curve evidenced that a Stroke Panel >4 predicted the occurrence of death at 120 days [area under the curve: 0.62 (95 % CI 0.55–0.69], *p* = 0.002) with a sensitivity of 80 % and a specificity of 42 % (Fig. 1).

Death was seen in 68/161 patients with an increase of biomarkers (42.2 %) compared with 17 of the 83 patients without an increase of biomarkers (20.5 %). Multivariate regression analysis found that a high NIHSS score on admission (OR 1.08; 95 % CI 1.02–1.14, *p* = 0.005), history of ischemic heart disease (OR 2.2; 95 % CI 1.1–4.5, *p* = 0.029), an increase of PCR (OR 2.8; 95 % CI 1.4–5.3, *p* = 0.002) and a Stroke Panel >4 (OR 3.1; 95 % CI 1.5–6.2, *p* = 0.002) were associated with adverse outcome (Table 2).

**Table 2:** Results of multivariate analysis (mortality at 120 days was the dependent variable)

	*P*	Odds ratio	95,0% Confidence IntervaI for OR	
			Lower	Upper
Sex M	,231	,672	,351	1,288
Age	,564	,993	,970	1,017
History of TIA	,967	,965	,182	5,124
Recurrent stroke	,474	,744	,331	1,672
Hypertension	,318	1,395	,726	2,681
Diabetes mellitus	,869	1,060	,532	2,111
Ipercholesterolemia	,875	,923	,344	2,480
Ipertrigliceridemia	,891	1,099	,286	4,224
Current smoking	,243	2,003	,625	6,424
Ischemic heart disease	,029	2,212	1,085	4,509
Alcohol	,884	,863	,118	6,304
NIHSS on admission	,005	1,080	1,023	1,140
Atherosclerosis	,829	1,121	,399	3,148
Stroke Panel >4	,002	3,078	1,511	6,271
Hyperhomocisteinemia	,559	1,229	,616	2,454
PCR increase	,002	2,795	1,470	5,312
Vitamin B12	,734	,875	,405	1,888
Ischemic stroke	,485	,733	,307	1,751

### Independent predictors of an increase of biomarkers

The characteristics of patients with and without an increase of inflammatory biomarkers are summarized in Table 3.

**Table 3:** Characteristics of the patients with Triage Stroke Panel > 4

Risk factors	Stroke Panel >4 (*n* = 161)	Stroke Panel ≤4 (*n* = 83)	*P*
Age (mean)	72.7 ± 13.3	73.7 ± 13.5	0.5
Sex male	92 (57.1 %)	39 (47.0 %)	0.1
Hypertension	94 (58.3 %)	61 (73.5 %)	0.025
Diabetes mellitus	43 (26.7 %)	28 (33.7 %)	0.3
Hypercholesterolemia	25 (15.5 %)	12 (14.4 %)	1.0
Hypertrygliceridemia	11 (6.8 %)	5 (5.9 %)	1.0
Current smoking	14 (8.7 %)	6 (7.2 %)	0.8
Stroke recurrence	27 (16.8 %)	17 (20.5 %)	0.4
Ischemic heart disease	49 (30.4 %)	30 (36.1 %)	0.4
Atrial fibrillation	7 (4.3 %)	3 (3.6 %)	1.0
Carotid atherosclerosis	16 (9.9 %)	11 (13.2 %)	0.6
Non-lacunar stroke	85 (52.8 %)	35 (42.2 %)	0.1
NIHSS on admission (mean)	8.7 ± 6.2	9.0 ± 6.2	0.7
Hyperhomocysteimenia	74 (45.9 %)	32 (38.5 %)	0.2
PCR increase	77 (47.8 %)	21 (25.3 %)	0.001
Mortality	68 (42.2 %)	17 (20.5 %)	0.001

At logistic regression analysis, an increase of biomarkers was independently predicted by an increase of PCR on admission (OR 2.9, 95 CI 1.4–6.0, *p* = 0.003).

## Discussion

In this study, we found that an increase of biochemical markers as BNP, D-Dimers, MMP-9, S and 100β tested with a Triage Stroke Panel (>4) was correlated with mortality at 120 days from stroke onset.

According to a significant increase of markers of inflammation, thrombosis and cellular death as well as myelin damage within 24 h from stroke onset this point-of-care immunoassay (Triage Stroke Panel®) has been developed to improve the reliability of the clinical diagnosis of stroke. Unfortunately, neither one marker nor combination of all markers was of significant benefit in acute stroke diagnostics. DWI-MRI is still the procedure with the highest diagnostic quality in case of acute cerebral ischemia [8]. Although the diagnostic accuracy of the current panel is clearly imperfect, the Triage Stroke Panel seems to have prognostic implications. Until now no study had been focusing on the MMX value as an index of poor prognosis in 120 days and the different parameters, separately studied, did not provide the same results [14–17]. Interestingly, aside from S100β, which is a marker of astrocyte activation, the biomarkers in this stroke panel are not specific to central nervous system tissues. Thus, although the majority of the markers used in this panel are not specific for cerebral ischemia, they represent different components of the ischemic cascade and when used in conjunction, they provide complementary information in the prognosis of stroke. In fact, these biomarkers may be elevated in the setting of medical comorbidities. For example, elevated levels of BNP are associated with congestive heart failure [18] and D-dimer [19] is elevated in any clinical circumstance in which both clot formation and subsequent fibrinolysis are increased, including deep venous thrombosis, pulmonary embolism, disseminated intravascular coagulation, acute myocardial infarction, surgery, and trauma. For these reasons, the Triage Stroke Panel could have more efficacy in defining stroke outcome than stroke diagnosis and its results should not be used as absolute evidence for cerebral ischemia. Patients who are experiencing a heart attack, patients who are candidates for renal dialysis or have had renal dialysis and patients with heart failure may have elevated MMX results. Results should be interpreted along with clinical findings and other test results. In 17 of the 83 patients without an increase of biomarkers, we have not observed the ischemic cascade and a rise of MMX, because the size of the lesion was modest and there were no significant comorbidities able to increase the values of biomarkers. Death has probably occurred for other causes or due to a re-stroke. Although ROC curve gave significant results, sensitivity and specificity values were minimally significant. Other limitations that it is necessary to mention are the residual confounding, the source of collection of data and the lack of MRI data.

## Conclusions

The current study demonstrates the feasibility of incorporating a biomarker-based point-of-care algorithm with readily available clinical data to aid in the early evaluation of outcome in patients with stroke. In the patient’s management, this data can greatly influence the approach to rehabilitation therapy, because this test can show us which patients will benefit most from this therapy since the early degree of the disease.
